# A Comparison of the Outcomes of Transarterial Chemoembolization and Transarterial Radioembolization in the Management of Neuroendocrine Liver Metastases in Adults: A Systematic Review

**DOI:** 10.7759/cureus.40592

**Published:** 2023-06-18

**Authors:** Nishok Victory Srinivasan, Sathish Venugopal

**Affiliations:** 1 General Surgery, California Institute of Behavioral Neurosciences & Psychology, Fairfield, USA; 2 Neurology, California Institute of Behavioral Neurosciences & Psychology, Fairfield, USA

**Keywords:** prognostic markers, overall survival (os), transarterial radioembolization, transarterial chemoembolization (tace), intra-arterial therapy, unresectable, liver metastases, hepatic metastases, primary neuroendocrine tumor

## Abstract

The purpose of this article is to review the existing English scientific literature and determine the superior modality between transarterial chemoembolization (TACE) and radioembolization (TARE) in the treatment of neuroendocrine liver metastases (NELMs). To that end, we followed the Preferred Reporting Items for Systematic Reviews and Meta-Analyses (PRISMA) guidelines to search PubMed, the Cochrane Library, and Google Scholar. We identified 14 observational studies and no randomized controlled trials (RCTs) investigating the use of TACE or TARE to treat NELM. We used the Newcastle-Ottawa Scale to assess the risk of bias in these studies. We concluded that TACE and TARE appeared to have similar outcomes when comparing overall survival, progression-free survival, radiological response, symptomatic response, and the incidence of severe adverse events. Further large-scale RCTs are needed to identify the superior modality conclusively. We also identified several unique prognostic factors for overall survival, such as the neutrophil-lymphocyte ratio, volumetric multiparametric magnetic resonance imaging, serum albumin, alkaline phosphatase, and pancreastatin.

## Introduction and background

Neuroendocrine tumors (NETs) are indolent neoplasms that arise from amine precursor uptake and decarboxylation (AUPD) cells, predominantly in the aerodigestive tract but potentially anywhere in the body [1,2]. In 2017, a large-scale study determined that the annual incidence of NETs was 6.98 out of 100,000, with a 20-year limited-duration prevalence of 48 out of 100,000 in the United States [3]. And although NETs are relatively indolent, more than one-fifth of patients will have distant metastases, with the liver being the most common site (60-90% of small bowel NETs will develop liver metastases) [4,5]. Regardless of the primary tumor site, hepatic metastases of NETs are not only acknowledged as a significant negative prognostic factor [6,7] but also a predictor of worse quality of life (QOL) due to frequent episodes of carcinoid syndrome [3,8].

The management of neuroendocrine liver metastases (NELMs) is complicated. Somatostatin analogs (SSAs) can be used for symptomatic relief in patients with hormone-producing NETs and potentially limit tumor growth [9]. Surgical resection is the only truly curative option and carries the best long-term outcomes, but only 10% of NELM patients fulfill the eligibility criteria [10,11]. The diffuse bilobar/multifocal nature of the metastases, the presence of extrahepatic disease, and prohibitive patient comorbidities are the most common reasons barring them from surgery [12]. Traditional systemic chemotherapy has also failed to significantly affect disease control or improve overall survival. A relatively recent advancement for patients unamenable to surgery is locoregional therapy, or liver-directed therapy [1]. This includes intra-arterial therapies (IAT) such as transarterial embolization (TAE), chemoembolization (TACE), and radioembolization (TARE) with Yttrium-90 (Y-90), as well as radiofrequency/microwave ablation (RFA/MWA) [13].

IATs have been proven to improve local tumor control and overall survival, palliate symptoms, and treat multiple tumors concurrently [13,14]. However, despite incorporating IATs into the guidelines for NELM management, there is insufficient evidence to decide which IAT modality is superior. Studies directly comparing TACE and TARE are rare and limited by small sample sizes or single-institutional designs. This review aims to collate and compare the reported data on the outcomes of TACE and TARE in studies published from 2010 to 2022.

## Review

Methods

We conducted the literature search and review according to the guidelines in Preferred Reporting Items for Systematic Reviews and Meta-Analyses (PRISMA) [15].

Eligibility Criteria

We included observational studies and randomized control trials (RCTs) reporting the treatment of hepatic metastases of neuroendocrine tumors (NETs) using transarterial chemoembolization (TACE) or radioembolization (TARE) for this review. Only studies published from 2010 to 2022 with a population greater than 80 adult patients (>18 years) were included.

Studies about the use of TACE or TARE for the treatment of other tumor types were excluded. Case reports and existing traditional/systematic reviews were also excluded.

Objectives

The primary endpoints of this review are median overall survival (OS) and radiological response to treatment, as evaluated by the Response Evaluation Criteria in Solid Tumors (RECIST) guidelines. The secondary outcomes are symptomatic response, five-year survival, severe adverse events (grade 4 and above by the Common Terminology Criteria for Adverse Events (CTCAE)), and prognostic factors influencing overall survival after TACE and TARE.

Search Strategy and Study Selection

We conducted a literature search on September 5, 2022, using PubMed, the Cochrane Library, and Google Scholar databases. The complete search strategy is detailed in Table 1. No additional filters were used.

**Table 1:** Search strategy MeSH: Medical Subject Headings; TARE: transarterial radioembolization; TACE: transarterial chemoembolization; NET: neuroendocrine tumor

Database	Search Strategy	Result
PubMed	("Embolization, Therapeutic"[Majr] OR Radioembolization OR TARE OR Transarterial radioembolization OR Chemoembolization OR Transarterial chemoembolization OR TACE OR "Chemoembolization, Therapeutic"[Majr]) AND ("Neuroendocrine tumors"[Majr] OR NET OR Neuroendocrine tumor) AND (Hepatic OR Liver OR "Liver"[Majr])	887 articles
Cochrane Library	(MeSH descriptor: [Embolization, Therapeutic] explode all trees OR Radioembolization OR TARE OR MeSH descriptor: [Chemoembolization, Therapeutic] explode all trees OR Chemoembolization OR TACE) AND (MeSH descriptor: [Neuroendocrine tumors] explode all trees OR NET OR Neuroendocrine tumor) AND (MeSH descriptor: [Liver] explode all trees OR Hepatic OR Liver)	64 articles
Google Scholar	(Embolization OR Radioembolization OR TARE OR Transarterial radioembolization OR Chemoembolization OR Transarterial chemoembolization OR TACE) AND (NET OR Neuroendocrine tumor) AND (Hepatic OR Liver)	100 articles

**Figure 1:**
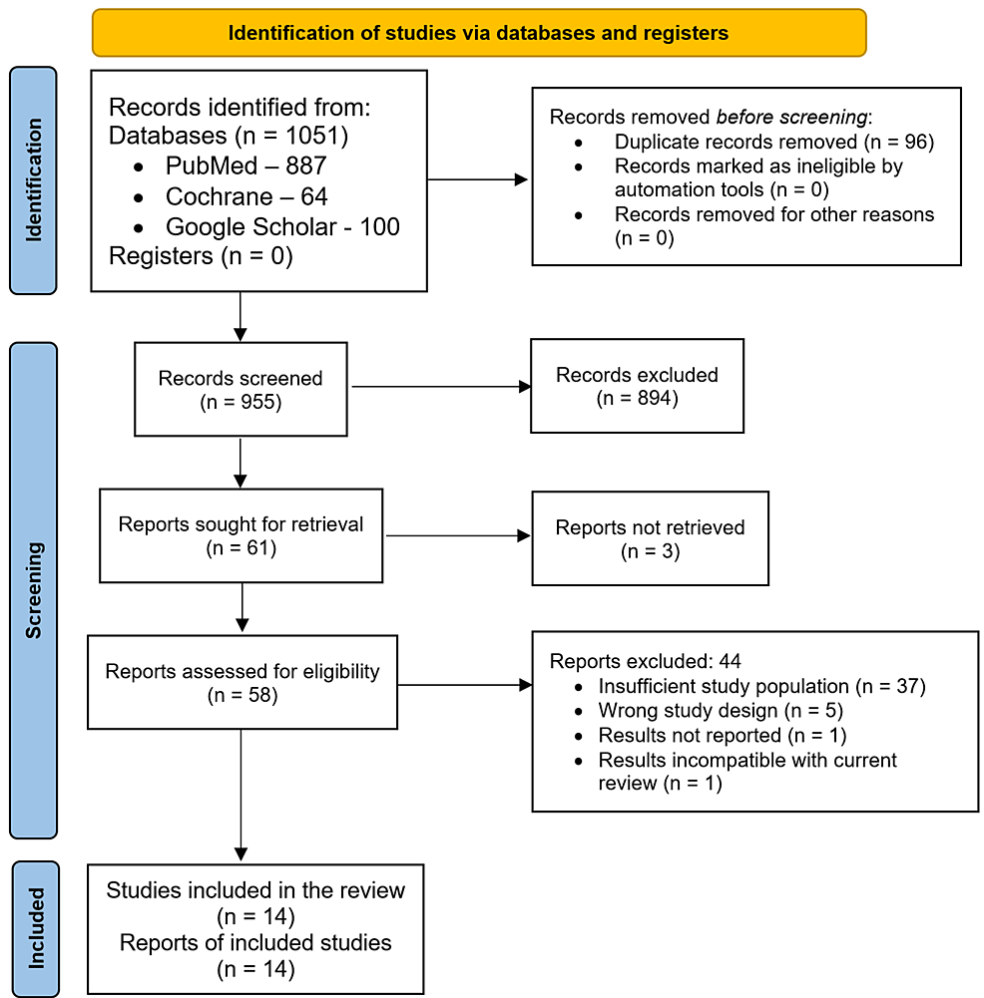
Study selection: PRISMA flowchart PRISMA: Preferred Reporting Items for Systematic Reviews and Meta-Analyses

A total of 1051 articles were obtained by executing the search strategy. These publications were then screened by their titles and abstracts using Rayyan, a screening tool for systematic reviews [16]. We retrieved the full texts of the studies shortlisted in this manner and included the studies that satisfied the inclusion criteria in our review. No studies were included from other sources. The PRISMA flowchart details the screening process (Figure 1).

Quality Assessment

We used the Newcastle-Ottawa Scale (NOS) to assess the quality of observational studies [17]. NOS groups study into low, moderate, or high risk of bias (corresponding to nine stars, seven or eight stars, and six or fewer stars, respectively). We planned to use the Cochrane Risk of Bias assessment tool for randomized control trials, but none were included in the final list.

Results

Search Results

From a total of 1051 articles obtained by executing our search strategy, we shortlisted 61. We were unable to acquire the full text of three articles. The full texts of the other 58 articles were scrutinized, and we were left with 14 articles after applying our inclusion and exclusion criteria. All the studies were observational (13 retrospectives and one prospective). A total of 2375 patients were enrolled in these studies; 1773 underwent TACE and 602 underwent TARE. 1043 patients (43.9%) were women; one study did not report the division of the study population by gender [18]. Most studies reported the outcomes of either TACE or TARE; only two studies compared TACE and TARE directly [19,20].

Study Characteristics

The details of the included studies are elaborated in Table 2 and Table 3, respectively. Minh et al. and Egger et al. are the two studies directly comparing the outcomes of TACE and TARE [19,20].

**Table 2:** Characteristics of TACE studies TACE: transarterial chemoembolization; NET: neuroendocrine tumor; NEC: neuroendocrine carcinoma; NELM: neuroendocrine liver metastases; STZ: streptozotocin; ECOG: Eastern Cooperative Oncology Group; IAT: intra-arterial therapy

Year of Publication	Primary Author	Journal	Study Design	Single or Multiple Centers	Sample Size	Patient Presentation
2010	Dong [21]	Medical Oncology	Retrospective Observational	Single Centre	123	Patient with unresectable metastatic NETs to the liver, treated with chemoembolization
2013	Arresse [22]	Annals of Surgical Oncology	Retrospective Observational	Single Centre	192	Consecutive patients with NEC metastases to the liver
2015	Onesti [23]	Journal of Gastrointestinal Surgery	Retrospective Observational	Single Centre	109	Patients with unresectable hepatic metastases of NET that underwent TACE
2016	Dhir [24]	Annals of Surgical Oncology	Retrospective Observational	Single Centre	91	Patients with unresectable NELM that underwent TACE with STZ
2016	Makary [25]	Journal of Vascular and Interventional Radiology	Retrospective Observational	Single Centre	177	Patients with histologically confirmed NET liver metastases that underwent TACE
2017	Minh [19]	European Radiology	Retrospective Observational	Single Centre	148	NELM patients with unresectable symptomatic or progressive hepatic-dominant disease and ECOG score <=2
2018	McDermott [26]	Journal of Surgical Research	Retrospective Observational	Single Centre	262	Patients with hepatic metastatic neuroendocrine cancer who underwent TACE
2018	Strosberg [1]	Annals of Surgical Oncology	Retrospective Observational	Single Centre	188	Consecutive patients with hepatic metastatic NET who underwent TACE
2019	Luo [2]	European Radiology	Retrospective Observational	Single Centre	84	Patients with NELM that underwent TACE
2020	Egger [20]	Journal of the American College of Surgeons	Retrospective Observational	Multicenter	197	Patients with hepatic NELM that underwent IAT (TACE or TARE)
2021	Touloupas [9]	Cancers	Retrospective Observational	Single Centre	202	Patients treated with TACE for NET liver metastases

**Table 3:** Characteristics of TARE studies TARE: transarterial radioembolization; TACE: transarterial chemoembolization; Y-90: yttrium 90; NELM: neuroendocrine liver metastases; NET: neuroendocrine tumor; NEN: neuroendocrine neoplasia; ECOG: Eastern Cooperative Oncology Group; IAT: intra-arterial therapy; RESiN: radiation-emitting SIR-spheres in non-resectable liver tumors; BMC: BioMed Central Ltd.

Year of Publication	Primary Author	Journal	Study Design	Single or Multiple Centers	Sample Size	Patient Presentation
2017	Minh [19]	European Radiology	Retrospective Observational	Single Centre	44	NELM patients with unresectable symptomatic or progressive hepatic-dominant disease and ECOG score <=2
2018	Tomozawa [27]	Journal of Vascular and Interventional Radiology	Retrospective Observational	Single Centre	93	Patients with NET liver metastases treated with Y-90 TARE
2019	Braat [18]	Cardiovascular and Interventional Radiology	Retrospective Observational	Multicenter	244	Patients with hepatic metastases of NEN of any origin, with baseline and 3 ± 1.5 months follow-up cross-sectional imaging.
2020	Egger [20]	Journal of the American College of Surgeons	Retrospective Observational	Multicenter	51	Patients with NELM that underwent IAT (TACE or TARE)
2022	Wong [28]	BMC Cancer	Prospective Observational	Multicenter	170	Patients with NELM enrolled in the RESiN registry

Demographic Details of the Study Population

In the reviewed studies, 1773 patients (873 females) underwent TACE. 441 patients had a pancreatic primary, while 947 had a gastrointestinal or bronchial primary. 981 patients were symptomatic (two studies did not report this statistic [2,19]). Further details can be found in Table 4.

**Table 4:** Demographic details: TACE TACE: transarterial chemoembolization; GIT: gastrointestinal tract; C-TACE: conventional transarterial chemoembolization; DEB-TACE: drug-eluting bead transarterial chemoembolization ^A^ – Median (range) ^B^ – Mean ± standard deviation ^C^ – Median (interquartile range)

Primary Author	Sample Size	Follow-up Period (Months)	Age (Years)	Female	%	Symptomatic Patients	%	Primary Tumor Location				Tumor Burden >50%	%	Bilobular	%	Extra-hepatic Metastases	%
								Pancreas	%	GIT/Lungs	%						
Dong [21]	123	38.4	56 (14.3-85.5)^ A^	59	47	114	92.7	15	12.2	70	56.9	-	-	-	-	-	-
Arresse [22]	192	91	54 (16-87)^ A^	92	48	157	81.7	43	22	103	54	185	96	-	-	123	64.1
Onesti [23]	109	42.9	61 (20-78)^ A^	47	43	85	77.9	20	18.3	61	55.9	63	57.8	-	-	54	49.5
Dhir [24]	91	37	62.1 ± 7.1^ B^	49	54	50	55	22	24	55	60.4	23	25	78	85.7	42	46
Makary [25]	C-TACE - 78	-	59.8 ± 13^ B^	29	37.2	47	60.2	9	11.5	55	70.5	-	-	73	93.6	-	-
	DEB TACE - 99	-	59.3 ± 12^ B^	51	51.5	60	60.6	36	36.4	42	42.4	-	-	92	92.9	-	-
Minh [19]	C-TACE - 122	75.6	57.3 ± 2^ B^	57	46.7	-	-	44	36.1	78	63.9	40	32.8	109	89.3	31	25.4
	DEB TACE - 26		61.4 (56.1-66.6)	10	38.5	-	-	10	38.5	16	61.5	8	30.8	24	92.3	8	30.8
McDermott [26]	262	36	57 (12-85)^ A^	142	45%	134	51	67	26	126	48	-	-	-	-	-	-
Strosberg [1]	188	37	58 (20-85)^ A^	98	52.1	144	76.6	43	22.9	94	50	146	77.8	178	94.6	60	31.9
Luo [2]	84	28	59 ± 12^ B^	33	39	-	-	34	40	28	33	-	-	-	-	32	38
Egger [20]	197	34	60 (51-67)^ C^	105	53.3	115	58.4	46	23.4	97	49.2	-	-	110	57.6	-	-
Touloupas [9]	202	98.4	60 ± 13^ B^	101	50	75	37	52	26	122	61	66	32	-	-	125	63
Total	1773			873	49.2	981	55.3	441	24.9	947	53.4	531	29.9	664	37.4	475	26.8

On the other hand, a total of 602 patients (170 female) underwent TARE. 168 patients had a pancreatic primary, while 349 had a gastrointestinal or bronchial primary. Only two studies, Braat et al. and Egger et al. reported symptoms; 159 out of 295 patients were symptomatic [18,20]. Detailed information can be found in Table 5.

**Table 5:** Demographic details: TARE TARE: transarterial radioembolization; GIT: gastrointestinal tract ^A^ – Mean ± standard deviation ^B^ – Median (interquartile range)

Primary Author	Sample Size	Follow-up Period (Months)	Age (Years)	Female	%	Symptomatic Patients	%	Primary Tumor Location				Tumor Burden >50%	%	Bilobular	%	Extra-hepatic Metastases	%
								Pancreas	%	GIT/Lungs	%						
Minh [19]	44	75.6	60.8 ± 3.4^ A^	17	38.6	-	-	13	29.5	31	70.5	9	20.5	42	95.5	14	31.8
Tomozawa [27]	93	15	58.6 ± 13.7^ A^	47	50.5	-	-	27	29	66	70.9	26	28	29	31.1	32	34.4
Braat [18]	244	6	-	-	-	147	60.2	76	31.2	122	50	115	47.1	-	-	161	66
Egger [20]	51	34	60 (56-70)^ B^	32	62.7	12	23.5	16	31.4	27	52.9	-	-	28	58.3	-	-
Wong [28]	170	-	65.5 (56-73)^ B^	74	44%	-	-	36	21.10%	103	60.50%	-	-	77	45.30%	77	45.30%
Total	602			170		159		168	27.90%	349	57.90%	150	24.9	176	29.20%	284	47.20%

Outcomes

This review's primary and secondary endpoints are detailed in the tables below. Table 6 and Table 7 describe the outcomes following TACE and TARE, respectively.

**Table 6:** Outcomes of TACE TACE: transarterial chemoembolization; RECIST: Response Evaluation Criteria In Solid Tumors; C-TACE: conventional transarterial chemoembolization; DEB-TACE: drug-eluting bead transarterial chemoembolization * – Median ** – Grade 4 and above by Common Terminology Criteria for Adverse Events (CTCAE), or Clavien-Dindo Grade 3 and above ^A^ – Uses RECIST (N=) as the denominator ^B^ – Uses “Symptomatic patients” from Table 4 as the denominator

Primary Author	Sample Size	Overall Survival (Months) *	Hepatic Progression-Free Survival (Months) *	Symptomatic Response	%^B^	RECIST (N = )	Complete/Partial Response	% ^A^	Stable Disease	% ^A^	Progressive Disease	% ^A^	5-year Survival	Adverse Events/Complications **	%
Dong [21]	123	39.6	-	-	-	123	76	62	30	24	17	14	36%	-	
Arresse [22]	192	30	16	113	72	166	24	15	104	63	38	23	-	17	8.8
Onesti [23]	109	42.9	-	34	40	98	85	86.7	9	9.2	4	4.1	69.70%	12	11
Dhir [24]	91	44	29.7	27	54	83	39	47	34	41	10	12	40.80%	4	4.4
Makary [25]	C-TACE - 78	-	-	37	78.7	73	60	82.2	13	17.8	0	0	-	3	3.8
	DEB-TACE - 99	-	-	30	50	94	70	74.5	17	18.1	7	7.5	-	11	11.1
Minh [19]	C-TACE - 122	33.8	21.6	-	-	90	3	3.3	83	92.2	4	4.4	28.20%	0	0
	DEB-TACE - 26	21.7	14.6	-	-	23	1	4.3	21	91.3	1	4.3	10.30%	0	0
McDermott [26]	262	30.1	-	-	-	-	-	-	-		-		-	12	4.6
Strosberg [1]	188	37.8	-	102	70.8	155	35	22.5	96	61.9	4	2.6	-	15	7.9
Luo [2]	84	40	16	-	-	-	-	-	-		-		-	-	
Egger [20]	197	50.1	19.9	-	-	139	42	30	92	66	5	3.6	42%	18	9.2
Touloupas [9]	202	63.6	19.3	54	72	202	102	50	94	47	6	3		6	3
Total	1773			397	40.5	1107	537	48.5	593	53.5	96	8.7		98	5.5

**Table 7:** Outcomes of TARE TARE: transarterial radioembolization; RECIST: Response Evaluation Criteria In Solid Tumors * – Median # - Mean ± Standard deviation ** – Grade 4 and above by Common Terminology Criteria for Adverse Events (CTCAE), or Clavien-Dindo Grade 3 and above ^A^ – Uses RECIST (N=) as the denominator ^B^ - Uses “Symptomatic patients” from Table 5 as the denominator

Primary Author	Sample Size	Overall Survival (Months) *	Hepatic Progression-Free Survival (Months) *	Symptomatic Response (%)	RECIST 1.1 (N = )	Complete/Partial Response	%	Stable Disease	%	Progressive Disease	%	5-year Survival	Severe Adverse Events **	%
Minh [19]	44	23.6	11.2	-	36	0	0	32	88.9	4	11.1	18.50%	0	0
Tomozawa [27]	93	28 ± 11.8 ^#^	-	-	52	13	25	35	67	4	8	-	0	0
Braat [18]	244	31.2 ± 4.8 ^#^	-	116 (78.9)^ B^	116	33	28.5	73	62.9	10	8.6	-	0	0
Egger [20]	51	35.9	15.9	-	46	11%	24	27	59	8	17.4	35%	3	5.9
Wong [28]	170	33	25	-	99	44	36	39	32	16	13	46%	45	26.5
Total	602			116	349	101	28.9	206	59	42	12		48	7.9

**Figure 2:**
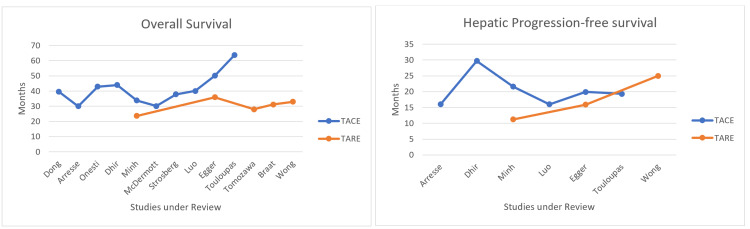
Overall and hepatic progression-free survival: TACE vs. TARE TACE: transarterial chemoembolization; TARE: transarterial radioembolization

**Figure 3:**
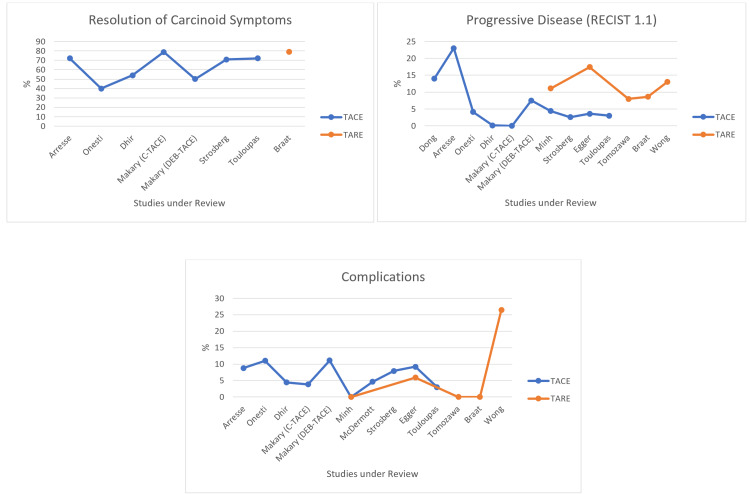
Other outcomes: TACE vs. TARE TACE: transarterial chemoembolization; TARE: transarterial radioembolization; RECIST: Response Evaluation Criteria In Solid Tumors; C-TACE: conventional transarterial chemoembolization; DEB-TACE: drug-eluting bead transarterial chemoembolization

Figure 2 and Figure 3 below depict the outcomes of TACE and TARE in graphical form. The results of the individual studies cannot be pooled or directly compared because the study populations cannot be matched. However, the graphs offer a crude comparison of the outcomes for ease of comprehension.

Minh et al. and Egger et al. were the only two studies that directly compared the outcomes of TACE and TARE [19,20]. Minh et al. reported significantly prolonged overall survival (OS) and hepatic progression-free survival (PFS) in patients undergoing conventional TACE (c-TACE) compared to TARE with Yttrium-90 (Y-90) (34 vs. 22.9 months, p = 0.032, and 21.6 vs. 11.2 months, p = 0.03, respectively). However, there was no significant difference in the radiological response to treatment (RECIST 1.1). On the other hand, Egger et al. reported no significant difference in the OS or PFS of TACE vs. TARE groups but described a significantly better radiological response (RECIST 1.1) with TACE (p = 0.0002).

Table 8 and Table 9 describe the prognostic factors influencing overall survival.

**Table 8:** Prognostic factors for overall survival following TACE (multivariate analysis) TACE: transarterial chemoembolization; NLR: neutrophil-lymphocyte ratio; RECIST: Response Evaluation Criteria In Solid Tumors; CR: complete response; PR: partial response; SSA: somatostatin analogues * - These studies reported that an increase in patient age, tumor burden, or percentage of hepatic involvement corresponded to a worse prognosis. Specific cut-off values were not reported.

Author	Negative Prognostic Factors	Positive Prognostic Factors
Dong [21]	Age*, Prothrombin time >13, Baseline serum albumin <3.5 mg/dL	-
Arresse [22]	Extrahepatic metastases	-
Onesti [23]	Preprocedural elevation of alkaline phosphatase	-
Dhir [24]	Age*, Tumor grade*, Hepatic tumor burden >75%	Serum chromogranin A <115 ng/ml
Minh [19]	Age >70 years, Tumor burden >50%, Presence of extrahepatic disease	-
McDermott [26]	Age >50 years, Pre-TACE NLR >4, 6 months post-TACE NLR > Pre-TACE NLR, Tumor grade 3	Resected primary tumor
Strosberg [1]	Preoperative serum pancreastatin >5000 pg/ml, Grade 3 tumor, Percentage of hepatic involvement*	Decrease in serum pancreastatin by >50% at any time after TACE
Luo [2]	Tumor volume >73 cm^3^	Baseline mean volumetric mean arterial enhancement >45%, Baseline mean volumetric mean venous enhancement >73%
Touloupas [9]	Age*, Progressive disease on RECIST, Grade 2 and 3 tumors, Hepatic tumor burden >25%	CR/PR on RECIST, Concomitant use of SSAs

**Table 9:** Prognostic factors for overall survival following TARE (multivariate analysis) TARE: transarterial radioembolization; NET: neuroendocrine tumor; NEC: neuroendocrine carcinoma; RECIST: Response Evaluation Criteria In Solid Tumors; DCR: disease control rate; ECOG: Eastern Cooperative Oncology Group * - This study reported that an increase in patient age corresponded to a worse prognosis. Specific cut-off values were not reported.

Author	Negative Prognostic Factors for Overall Survival	Positive Prognostic Factors
Minh [19]	Age*, Tumor burden >50%, Presence of extrahepatic disease	-
Tomozawa [27]	Extrahepatic metastases, Ascites	-
Braat [18]	Grade 3 NET/NEC, Intra-hepatic tumor load >75%, Presence of extrahepatic disease	DCR by RECIST 1.1
Wong [28]	ECOG >=2, Baseline ascites	Unilobar disease

Discussion

The traditional curative therapy for neuroendocrine tumors has always been surgical resection [10,11]. However, neuroendocrine liver metastases (NELMs) are only amenable to resection in approximately 10% of patients, and traditional systemic chemotherapy is mostly ineffective [1]. This leads to the need for other treatment modalities, such as intra-arterial therapies. IATs work on the concept of differential vascular supply; NELMs are highly vascular but derive 80-90% of their blood supply from the hepatic artery. In contrast, the portal vein predominantly supplies normal liver tissue. This means that therapeutic agents can be delivered to the tumor via the hepatic artery with minimal influence on the normal liver parenchyma [29].

Transarterial embolization (TAE) takes advantage of this concept by sending embolic agents directly into the branches of the hepatic artery supplying the tumor, cutting off its blood supply and causing ischemic necrosis. Transarterial chemoembolization (TACE) takes this a step further, adding targeted chemotherapy to the embolic agents and directly delivering the chemotherapeutic agents to the tumor, unlike systemic chemotherapy. Transarterial radioembolization (TARE) delivers microspheres of Yttrium-90 instead of embolic agents or drugs, applying radiation selectively to the tumor at a short range; it has been reported to have a lower incidence of adverse events and lower retreatment rates compared to TACE (which usually requires multiple treatment sessions) [19]. IATs are used in cases of unresectable hepatic metastases and may also be used as neoadjuvant therapy to reduce the tumor burden.

This review aims to compare the outcomes of TACE and TARE in the treatment of NELMs. The last systematic review on this topic was published in 2012, so we only included studies published between 2010 and 2022 [29]. As IATs become more accessible to healthcare providers and patients, knowing which option provides the best quality of care is essential. We analyzed 14 independent studies to provide an answer.

Our literature search of PubMed, the Cochrane Library, and Google Scholar generated a total of 1051 articles, from which we screened 61. Scrutinizing the full texts and applying the inclusion/exclusion criteria left us with 14 articles eligible for our review: 13 retrospective observational studies and one prospective observational study, with no randomized control trials. These 14 studies encompassed 2375 patients with NELM, of whom 1773 underwent TACE and 602 underwent TARE. Only two studies directly compared the outcomes of TACE and TARE [19,20]; three studies only discussed TARE [18,27,28]; while the remaining nine discussed TACE only [1,2,9,21-26].

Overall Survival (OS)

The duration of overall survival following TACE ranged from 21.7 to 63.6 months, with a median of 39.6 months. OS after TARE ranged from 23.6 to 35.9 months, with a median of 31.2 months (see Table 6, Table 7, and Figure 2). In one study on TACE, Makary et al. did not report OS [25]. Minh et al. reported a significantly extended OS with TACE over TARE (34 months vs. 22.9 months, p = 0.032) [19]. Egger et al., on the other hand, concluded that there were no significant differences in OS between the TACE and TARE cohorts [20]. The OS seemed similar across TACE and TARE studies in this review, with Touloupas et al. being the only outlier [9]. No studies reported a significant difference in overall survival between different primary tumor locations.

Arresse et al. reported that patients with extrahepatic disease (EHD) had significantly lower overall survival than patients without EHD (28 months vs. 62 months). The two cohorts had similar survival rates until the two-year mark, after which patients with EHD deteriorated rapidly [22]. Dhir et al. also reported a novel finding: post-progression median survival is approximately 18 to 24 months from the incidence of post-TACE progression [24].

Hepatic Progression-Free Survival (PFS)

PFS was analyzed by comparing baseline and follow-up imaging (CT or MRI) to detect the progression of the tumor. It ranged from 14.6 to 29.7 months (median 19.3 months) and 11.2 to 25 months (median 15.9 months) after TACE and TARE, respectively (Table 6, Table 7, and Figure 2). As with OS, Minh et al. reported a significant difference in PFS between the TACE and TARE cohorts (21.6 vs. 11.2 months, p = 0.03), while Egger et al. reported no significant difference [19,20]. Five studies in the TACE group and two in the TARE group did not report PFS. As seen in Figure 2, PFS across TACE and TARE studies seemed similar. Primary tumor location did not influence PFS in any study.

Resolution of Symptoms

Seven studies reported the number of patients exhibiting symptoms due to NELMs and the number of patients who resolved their symptoms post-therapy. A total of 765 patients were reported as symptomatic, with 618 in the TACE cohort and 147 in the TARE cohort. Of those, 513 patients exhibited a symptomatic response to treatment (397 after TACE, 116 after TARE). TACE and TARE appear to be equally effective in resolving the symptoms of functional NELMs (Figure 3). However, only one study in the TARE group reported the resolution of symptoms [18], so this comparison is relatively unreliable.

Dong et al. discovered that the level of hormone production by the NET did not correlate with symptoms because most tumors produced non-functional hormones (pancreatic polypeptide was the most common, beating out serotonin) [21].

Radiological Response to Treatment

The RECIST 1.1 guidelines assessed the radiological response to treatment. mRECIST is considered more accurate than RECIST 1.1 ([30]), but both are equally effective in monitoring disease progression. We used RECIST 1.1 because it was available in most of the studies under review. The disease control rate (DCR) ranged from 77.1% to 100% in TACE studies and from 82.6% to 92.3% in TARE studies, which are very similar. On the other hand, the rate of progressive disease (PD) ranged from 0% to 23% in TACE studies and from 8% to 17.4% in TARE studies. As seen in Figure 3, TARE seems to have an overall higher PD rate than TACE. Minh et al. and Egger et al. again report opposing conclusions: Egger et al. report a significantly better radiological response after TACE (p = 0.0002), while Minh et al. reports no significant difference (p = 0.56). Despite being a relatively objective evaluation of the response to treatment, RECIST is an unreliable endpoint in this review. The required follow-up imaging and assessment were performed at variable times in different studies and even within the same study.

Adverse Events or Complications

We recorded severe adverse events as post-procedural complications grade 4 and above by CTCAE or grade 3 and above by Clavien-Dindo classification. The incidence was low overall, with a maximum of 11.1% following TACE. TARE also had a minimal complication rate, with a maximum of 5.9% after excluding an outlier (Wong et al., with a complication rate of 26.5%) [28]. We could not discern why Wong et al. reported such a disproportionately elevated complication rate. Excluding Wong et al., TARE appears to have an overall lower rate of adverse events than TACE.

Prognostic Factors for Overall Survival

The percentage of hepatic involvement (i.e., hepatic tumor burden) is the most frequently mentioned negative prognostic factor reported in six studies. Advancing patient age and extrahepatic disease were the second most common, with five mentions each. Tumor grade came in third with four mentions (Table 8 and Table 9).

Dong et al. found that elevated prothrombin time and low serum albumin identified patients with poorer chances of survival, possibly because they are both markers of liver function and, thus, malnutrition [21]. Malnourished patients would be susceptible to post-procedural complications and disease progression. Arresse et al. compared patients with extrahepatic disease (EHD) vs. those without EHD and found that overall survival was significantly lower in patients with EHD, but the rate of complications was independent of EHD and dependent on hepatic tumor involvement (liver replacement) instead [22]. They also concluded that despite the lower overall survival, patients with EHD still lived long enough (median survival >two years) to benefit from TACE.

Onesti et al. studied the use of serum alkaline phosphatase (ALP) as a predictor of worse outcomes [23]. They opined that in patients with hepatic metastases, elevated serum ALP reflected the leakage of ALP from damaged hepatocytes, making serum ALP an indicator of hepatic damage. Multivariate analysis proved significantly worse OS in patients with elevated preoperative ALP (HR = 4.7, p = 0.001) [23]. Dhir et al. identified a similar association between serum chromogranin A (CgA) and hepatic tumor involvement; further analysis proved that there was a significant decrease in overall survival corresponding to high (>115 ng/ml) CgA [24]. This is particularly important as CgA is the most commonly tested tumor marker for NETs (despite levels varying with medications and diurnal variation).

McDermott et al. noticed that a pre-TACE neutrophil-lymphocyte ratio (NLR) of less than four corresponded to a higher overall survival than a pre-TACE NLR greater than four. Further analysis revealed that OS was lower if the post-TACE NLR at six months was greater than the pre-TACE NLR, but the NLR in the immediate post-procedural period (one day and one-week post-TACE) was not significant. Here, NLR is a marker of inflammation correlated to tumor burden [26].

Strosberg et al. focused on using serum pancreastatin, a split product of chromogranin A and a known biomarker for NETs, to predict survival [1]. Pancreastatin is less variable than CgA and can be measured by a standardized assay [31]. Strosberg et al. discovered that a pre-TACE serum pancreastatin greater than 5000 pg/ml was predictive of significantly lower overall survival (22.1 vs. 58.5 months, p < 0.001) and remained significant on multivariate analysis. They also found that a decrease in serum pancreastatin levels by 50% or more at any point after TACE was associated with a significant increase in overall survival. Patients with increasing post-TACE serum pancreastatin, on the other hand, were more likely to have progressive disease (p = 0.002) and need repeat TACE (p = 0.009) [1]. Arresse et al. also reported that a 20% or greater decrease in serum pancreastatin corresponded to prolonged overall survival [22]. Earlier studies had concluded that there was an association between serum pancreastatin and overall survival in patients with NELM [32,33]; Strosberg and Arresse confirmed that the association held true after locoregional therapy (TACE). Strosberg et al. suggest that serum pancreastatin can be monitored in addition to radiographic assessment to assess treatment response and disease progression [1].

Luo et al. investigated the prognostic value of volumetric magnetic resonance (MR) imaging in their study; as a non-invasive imaging biomarker, volumetric MR is associated with decreased costs and morbidity. As mentioned earlier, the rationality of IATs is based on the vascularity of the tumor, which can be quantified by measuring enhancement on radiological imaging. Previous studies have discovered a correlation between tumor enhancement and overall survival after TACE and TARE, with hyperenhancement corresponding to better outcomes and hypoenhancement with earlier disease progression [34-36]. Luo et al. employed a quantitative method and concluded that baseline mean arterial and venous enhancements were independent predictors of overall survival [2], as seen in Table 8. On the other hand, the apparent diffusion coefficient (ADC) did not correlate with therapeutic outcomes.

Tumor volume was also determined to be an independent predictor of overall survival. Luo et al. proposed it be used as an objective measure of hepatic tumor burden/involvement, a common predictor of poorer outcomes. Despite the frequent mention of hepatic tumor burden as a prognostic factor, it is assessed by a semi-quantitative visual estimation reproducible between observers in only 58% of cases [37]. In contrast, the tumor volume automatically generated during volumetric MR imaging is an objective measurement and an independent predictor of overall survival [2].

Limitations

All but one of the reviewed studies were retrospective and non-randomized, with all the associated limitations. The majority were also monocentric (only three multicentric studies) with relatively small study populations, limiting the external validity of the results. The study cohorts included primary tumors of various origins, which may confound the results. The tumor grades were not always reported and had to be extrapolated from pathology reports, introducing uncertainty, especially in differentiating grade 1 and grade 2 tumors. The assessment of symptoms, the symptomatic response, and the incidence of adverse events are all subject to recall bias.

## Conclusions

In summary, both transarterial chemoembolization (TACE) and radioembolization (TARE) are effective therapeutic options for neuroendocrine liver metastases (NELMs). We reviewed overall survival, progression-free survival, radiological response, symptomatic response, and severe adverse events and found similar results comparing TACE and TARE. The choice of the superior treatment modality, however, remains inconclusive. TARE and TACE appear to have comparable outcomes, with their advantages and disadvantages. Further trials with larger, multicentric, randomized study populations directly comparing TACE and TARE are necessary to conclusively declare the superior modality.

Our review also noted several effective prognostic indicators that may have been overlooked. In addition to the well-known chromogranin A, serum alkaline phosphatase (ALP) and pancreastatin proved to be effective and potentially more accurate prognostic indicators. Further studies will be required to determine which one is most effective in clinical practice.
